# Premature ejaculation: challenging new and the old concepts

**DOI:** 10.12688/f1000research.12150.1

**Published:** 2017-12-04

**Authors:** Odunayo Kalejaiye, Khaled Almekaty, Gideon Blecher, Suks Minhas

**Affiliations:** 1 Department of Andrology, University College London Medical School, London, W1G 8PH, UK; 2 Urology Department, University of Tanta, Tanta, Egypt

**Keywords:** Premature ejaculation, Treatment, Aetiology, Diagnosis, Physiology

## Abstract

Premature ejaculation remains a difficult condition to manage for patients, their partners, and the clinician. Whilst prevalence rates are estimated to be 20–40%, determining a diagnosis of premature ejaculation is difficult, as the definition remains both subjective and ill-defined in the clinical context. As our understanding of the ejaculatory pathway has improved, new opportunities to treat the condition have evolved with mixed results. In this review, we explore some of these controversies surrounding the aetiology, diagnosis, and treatment of this condition and discuss potential novel therapeutic options.

## Introduction

Premature ejaculation (PE) is a complex and poorly understood condition which can be difficult to manage for both the clinician and the patient. In this review, we will challenge some of the accepted theories and treatments associated with this condition and explore the current management options and potential treatments.

## Does the concept of early ejaculation exist?

Mammals ejaculate quickly to enhance their ability to procreate with multiple partners and spread their genetic material. In humans, sexual intercourse not only allows us to fulfil our reproductive potential but also has important effects on our quality of life. As boys reach puberty and start engaging in sexual activity, their time to ejaculation and their perceived control over their ejaculation change over time ^1^. We accept this to be a normal part of sexual maturity; however, we may define the same features as a sign of PE in an adult. In addition, ejaculation control and time are dependent on the male, his partner, and the sexual situation ^2– 4^. Early ejaculation is not necessarily always associated with lack of sexual enjoyment, and the perception of a sexual dysfunction secondary to PE may be heavily influenced by social factors ^5– 7^. We also know that social media, cultural attitudes, and abstinence can all affect sexual function ^5– 7^. Therefore, defining PE as a pathological condition may not necessarily be correct. We should use these concepts to challenge whether PE truly exists or whether it has arisen as a result of men having unrealistic concepts of sexual function which are based on social conditioning, especially the use of pornography. PE is a multimillion-dollar industry and therefore the pharmaceutical industry must be challenged as to whether medicalising PE benefits our patients or whether they would be best served with psycho-sexual counselling ^1^. In addition, the literature rarely assesses or medicates the female partners.

## Prevalence

PE is estimated to affect 20–40% of the general population, although only 4–5% may fulfil the International Society of Sexual Medicine (ISSM) definition for this ^8– 13^. Nathan *et al*. reported a prevalence of 35% based on the *Diagnostic and Statistical Manual of Mental Disorders*, third edition, Text Revision (DSM-III-TR) definition ^3^. In an Italian telephone survey, 14.7% of the calls were regarding PE and peak age was 26–35 years ^14^. Women called less frequently about PE. Similar results were obtained from a study on subjects contacting a UK-based sexual advice charity by email or telephone ^2^. Although most visitors to the charity’s website were from the UK, significant proportions were from the USA, Singapore, Canada, and India. PE was reported by 16% contacting the charity, and the peak age in men was 31–40 years old ^2^. PE was more commonly reported by men outside the UK, especially from the Indian subcontinent. The huge variability in the reported prevalence is due to the different methods with which PE is defined, which include questionnaires, self-reports, and several expert panel definitions ^8– 11^. Despite the variability in reported prevalence rates, a consistently lower proportion of men seek treatment ^15, 16^. This may reflect embarrassment and the perceived stigma attached to the condition.

## Physiology

Ejaculation is a normal part of male sexual function and consists of two coordinated neurological reflexes: emission and ejection ^17^. Orgasm is separate but occurs simultaneously ^18^. Emission involves contractions of the seminal vesicles and prostate and results in expulsion of sperm and seminal fluid into the posterior urethra ^19^. This is mediated by the sympathetic nervous system (T10–L2). Ejection involves the pulsatile contractions of the bulbocavernosus and pelvic floor muscles with relaxation of the external urinary sphincter ^19^. This is mediated by somatic nerves (S2–4) and involves the sympathetic nervous system with limited voluntary control ^19^. These reflexes are stimulated by sensory input from nerve endings in the glans penis, which are relayed via the sacral spinal cord to the sensory cerebral cortex ^17^. The pathway involves central serotonergic and dopaminergic neurons with secondary involvement of cholinergic, adrenergic, oxytocinergic, and gamma aminobutyric acid (GABA) neurons ^19^.

Dopamine and serotonin are essential neurotransmitters; dopamine promotes seminal emission and ejaculation via D2 receptors, while serotonin is inhibitory via the serotonin receptors (5-HT) ^19^. Stimulation of the 5-HT _2c_ receptor results in ejaculatory delay in rats, whereas stimulation of the post-synaptic 5-HT _1A_ receptors results in shortening of ejaculatory latency time ^8, 19^. It has been postulated that PE is due to hyposensitivity of 5-HT _2C_ or hypersensitivity of 5-HT _1A_ receptors or both. The role of oxytocin is less well established and has been found to have a stimulatory effect on ejaculation in rat models and reduces ejaculatory latency times ^8, 20^. This may be as a result of modulating the action of the 5-HT _1A_ receptors ^21^.

The influence of hormones is less clear. Some animal models have suggested that cerebral dopamine and serotonin may interact with the hypothalamic-pituitary-thyroid axis ^22– 24^. Hyperthyroid rats were found to have shorter times to first ejaculation compared with controls ^25^. A small study by Carani *et al*. found a significant correlation between PE and hyperthyroid men, which fell dramatically, from 50 to 15%, following treatment ^26^. This would support a possible role for the thyroid hormones in ejaculation. However, other studies have suggested that there is a role only in acquired PE and this is a rare cause ^27, 28^.

A growing area in the aetiology of PE is its possible association with prostatitis. Sereponi *et al*. investigated the prevalence of chronic prostatitis in 46 men with PE and compared this group with an age-matched group of 30 healthy men ^29^. Prostatic inflammation and chronic bacterial prostatitis were found in 56.5% and 47.5% (respectively) of men with PE. In addition, 28.2% with PE were found to have one or more clinical symptoms of prostatitis. This study has been followed by several studies which have reviewed the effect of treating prostatitis on PE ^30, 31^. Treatment of prostatitis with a month of antibiotics based on culture sensitivities appears to increase ejaculatory latency times (2.6-fold) and control over ejaculation (83.9%) ^30, 31^. There was no recurrence of PE at a follow-up of 4 months ^30, 31^. Antibiotics appear to be most effective in men with acquired PE and where there are at least 19 pus cells per high-powered field in expressed prostate secretions ^31^. The mechanism remains poorly understood, but it has been suggested that prostatic inflammation may alter sensation and therefore the ejaculatory reflex ^32^. Although the evidence may still be growing, the assessment of the prostate appears to be a useful adjunct to the management of PE. However, as always, we must balance our use of antibiotics with our responsibility as antibiotic stewards in the growing international antibiotic crisis.

## How to define premature ejaculation

Early definitions of PE were criticised for their inconsistency and often having vague definitions subject to the clinician’s interpretation ^33, 34^. This has led to heterogeneous studies, adversely affecting both research and valid information regarding prevalence of the condition ^33, 34^. In 1980, the American Psychiatric Association published the DSM-III-TR, which has clearly defined PE albeit with many flaws ^35^. This definition ( Table 1) has been revised several times, and the current, fifth edition has corrected much of the earlier ambiguity ( Table 1) ^36^.

**Table 1.** DSM-V-TR definition of premature ejaculation ^36^.

• Persistent/recurrent ejaculation with minimal sexual stimulation before or shortly after penetration and before the person wishes it • Clinician should take into account factors affecting the duration of the excitation phase (for example, age, novelty of sexual partner/situation, and recent frequency of sexual activity) • Disturbance causes distress or interpersonal difficulty • Premature ejaculation not exclusively a result of direct effects of a substance (that is, opioid withdrawal)

DSM-V-TR, *Diagnostic and Statistical Manual of Mental Disorders*, fifth edition, Text Revision

This definition remains largely contentious with many ongoing criticisms. The absence of time criteria in the definition opens it up to misinterpretation by clinicians. This leads to inconsistencies in data collection and difficulty making direct comparisons between studies because of heterogeneous patient populations. This is illustrated by two studies using the DSM-V-TR definition (unchanged from version IV), which revealed significant overlap between the group defined as having PE and the group without PE ^37– 40^. A total of 13–25% of men with a diagnosis of PE according to the definition had intravaginal latency times of 4–25 minutes. In the study by Giuliano *et al*., 12.1% defined as not having PE had intravaginal latency times of less than 2 minutes ^39^.

A further criticism of the definition is that clinicians are asked to consider factors which may affect the excitation phase, such as age ^33^. However, there is no consistent evidence that age, novelty of partner, or relationship duration has any effect on ejaculatory latency times ^41, 42^. In a large global phone and email survey, men younger than 40 were significantly more likely to report PE ^2^. However, in a study of sexual behaviours and attitudes in the USA, the reverse was found ^4^.

The last major criticism is the absence of criteria for how frequently or the duration over which rapid ejaculation should occur before it may be defined as problematic ^33^. A man with frequent sexual engagement who occasionally experiences rapid ejaculation may not be bothered, whereas a man with infrequent sexual opportunity may be bothered by a single episode of rapid ejaculation. The definition does not clarify which subject should be diagnosed as having PE. The ISSM convened a panel of international experts to produce a new evidence-based definition (see Table 2 ^13, 43, 44^ for the current one) which would address most of the inconsistencies of earlier definitions.

**Table 2.** International Society of Sexual Medicine definition of premature ejaculation (2014) ^13, 43, 44^.

• Ejaculation which always or nearly always occurs prior to or within about 1 minute of vaginal penetration from first sexual experiences (lifelong premature ejaculation) or a clinically bothersome reduction in latency time, often to about 3 minutes or less (acquired premature ejaculation) • Inability to delay ejaculation on all or nearly all vaginal penetrations • Negative personal consequences such as distress, bother, frustration, and/or the avoidance of sexual intimacy

The main difficulties with this definition are the method of measuring the latency time and how to define a normal or abnormal time. The concept of the intravaginal ejaculatory latency time (IELT) was introduced by Waldinger *et al*. ^5, 45^ in 1994. This is the time from vaginal penetration to ejaculation. It is a useful measurement tool in PE studies and may be either estimated by the male or his partner or measured directly with the use of a stopwatch. Several studies have suggested an acceptable correlation between estimated times ^46, 47^. There was a tendency for men to overestimate their IELT ^6, 46, 47^. This would allow the use of estimated IELT measures in clinical practice instead of measured IELT which may not be practical for couples. However, the study by Pryor *et al*., which compared, estimated, and measured IELTs, based PE definition on the DSM-IV-TR criteria, which is an inherent flaw in the study ^46^. This suggests that the estimated and measured IELTs are equivocal and erroneous, and we may need to repeat studies using the ISSM definitions for PE. Two large international population studies have provided useful information on IELT in the general population ^5, 6^. Both revealed a positively skewed distribution in IELT, and median times were 5.4 minutes (range of 0.55–44.1) and 6 minutes (range of 0.1–52.7). There was significant variability across the countries involved. This may suggest that IELT definitions need to be adjusted depending on the ethnic mix of the study population. In addition, it provides a useful counselling tactic to reassure a male patient that he is actually within the normal distribution. However, studies which have analysed IELT in the general population have not included ethnically diverse populations. A study from the UK reported that the highest incidence of PE was in men from Asian and Islamic backgrounds, which may reflect a genetic or a cultural aetiology ^7^. More studies are required to define normal IELT in these populations.

The first study revealed a significant reduction in IELT with age:

- Age 18–30: IELT of 6.5 minutes
- Age 31–50: IELT of 5.4 minutes
- Age >50: IELT of 4.3 minutes ^5^

The use of the 0.5 and 2.5 percentile is an accepted method of defining disease within a skewed distribution ^48^. These two studies, therefore, have defined an abnormal IELT as being 1 minute ^5, 7, 46^. This has been incorporated within the ISSM PE definition ^44^ and has been supported in PE studies where 77–92% of men with PE generally ejaculate within 60 seconds ^47, 49, 50^.

The use of IELT is not without inherent problems. The data collected using IELT are usually based on either men with lifelong PE (LPE) or heterosexual men within stable relationships engaging only in vaginal penetrative sexual intercourse ^33^. This limits its general applicability to clinical populations. In addition, the act of measuring ejaculatory latency itself may affect sexual performance either positively or negatively. This is a factor which may be difficult to account for or quantify within clinical trials or medical practice.

The European Association of Urology (EAU) guidelines mention both definitions; however, it recommends that the diagnosis of PE include self-estimated IELT, perceived control, and interpersonal difficulty due to ejaculatory dysfunction ^51^. The American Association of Urology mentions the DSM-IV-TR in its discussion on PE definitions ^52^. However, it defines PE as “ejaculation that occurs sooner than desired, either before or shortly after penetration, causing distress to either one or both partners” ^52^.

## Validated questionnaires

Several validated questionnaires, which are mainly for use in clinical trials, have been developed ^53– 56^. Whilst they are useful adjuncts in clinical assessment and measuring the impact of treatment on PE, they are not without limitations, and they are used to assess LPE and men who have vaginal sexual intercourse ^57^. Currently, the EAU guidelines for PE recommend that both stopwatch-measured IELT and these types of questionnaire are not used in clinical practice ^51^.

### Premature Ejaculation Profile

This questionnaire, developed by Patrick *et al*., assesses four domains of PE as defined according to the DSM-IV-TR ^54, 57^:

- Perceived control over ejaculation
- Personal distress related to ejaculation
- Interpersonal difficulty related to ejaculation
- Satisfaction with sexual intercourse ^54^

Each domain is assessed by using a single question, and the response is rated on a five-point scale from 0–5; higher scores indicate better functioning. This is a validated questionnaire with good test-retest reliability and moderate/strong correlation with stopwatch measurements of IELT ^54, 57, 58^. Validation was in populations from Europe and the USA ^54^. It has been used extensively in observational and pharmacological trials ^54, 57^. The main advantages are that it is quick to complete and is useful as a measure of response to treatment ^58^. However, there are no validated cut-off values and it uses a single question to determine the effect within each domain ^57^. This would seem to be a major limitation when compared with the International Index of Erectile Function, where the erectile dysfunction (ED) domain score is determined by five questions and the scores are then divided into categories, which determine ED severity ^59^.

### Index of Premature Ejaculation

This questionnaire, developed by Althof *et al*., assesses three domains using 10 questions ^53, 54, 58^. It was validated in men with an IELT of not more than 1 minute ^57^. Validation studies revealed that the questionnaire has good validity and reliability ^57^. In this index, unlike in the PE Profile (PEP), each domain is assessed using several questions. Similar to the PEP, it does not have any validated cut-off values.

### Premature Ejaculation Diagnostic Tool

This is a screening questionnaire with five questions and no domains ^55, 57, 58^. Validation was based on focus and interviews of men from the USA, Germany, and Spain ^55^. Data were collected from men with PE defined according to the DSM-IV-TR and an IELT of not more than 2 minutes, men reporting PE, and men reporting no PE ^55^. Validation revealed good reliability and validity ^58^. The total score is from 0 to 25 ^55, 58^. A score of 8 indicates no PE, a score of 9–10 indicates possible PE, and a score of at least 11 indicates PE ^55^. This useful questionnaire allows a quick diagnosis of PE but does not provide objective evidence of response to treatment. McMahon *et al*. reported the prevalence of PE in the Asia-Pacific region by using self-reports and the Premature Ejaculation Diagnostic Tool (PEDT) ^11^. They found that only 40% with PE diagnosis using PEDT also reported having PE. This suggests limitations with both the questionnaire and the male definition of what constitutes PE ( Table 3) ^11, 55, 57, 58^.

**Table 3.** Premature ejaculation questionnaires ^11, 55, 57, 58^.

Questionnaire	Number of questions	Domain names	Advantages	Disadvantages
Premature Ejaculation Profile	4	Control over ejaculation Satisfaction with sexual intercourse Personal distress Interpersonal distress	Quick to complete Assess treatment outcome	Validation with *Diagnostic and Statistical* *Manual of Mental Disorders*, fourth edition, Text Revision (DSM-IV-TR) Single question to assess each domain No cut-offs
Interprofessional education	10	Control Sexual satisfaction Distress	Quick to complete Assess treatment outcome Validated in men with intravaginal ejaculatory latency time ≤1 minute	No cut-offs
Premature Ejaculation Diagnostic Tool	5	None	Screening questionnaire Cut-off values available Quick to complete	May not correspond with self-report of premature ejaculation

## Management options: historical options

The current EAU guidelines for the management of PE suggest the use of pharmacotherapy as first-line treatment ^51^. This includes either the short-acting dapoxetine (DPX) on demand or other off-label antidepressants such as daily selective serotonin reuptake inhibitors (SSRIs). Tramadol and topical local anaesthetics may be used as weak alternatives to SSRIs; phosphodiesterase-5 inhibitors (PDE5is) should be used only in men with concomitant ED ^51^. Behavioural therapy should be used with pharmacotherapy ( Table 4) ^60, 61^.

**Table 4.** Historic treatment options ^60, 61^.

Drug	Mechanism of action	IELT fold increase versus baseline	Side effects
Topical local anaesthesia	Reduces sensitivity of glans	EMLA: 5.6 TEMPE: 3–6.3	Partner hypoanaesthesia
Selective serotonin reuptake inhibitors (SSRIs)	Block axonal reuptake of serotonin from synaptic cleft Result in enhanced transmission and stimulation of 5-HT _2c_ receptors	Paroxetine: 2.68–11.6 Dapoxetine: 3.6–4.3 Citalopram: 1.2	Nausea Diarrhoea Headache Dizziness
Tramadol	Inhibition of 5-HT reuptake	3.3–5.6	Addiction Somnolence Pruritus Nausea Serotonin syndrome if used with SSRI
Phosphodiesterase-5 inhibitor (PDE5i)	Reduces contractile response of seminal vesicles and vas deferens	Sildenafil: 2.7–3.9 Vardenafil: 6.5	Headache Flushing

5-HT, 5-hydroxytryptamine; EMLA, eutectic mixture of local anaesthetics; IELT, intravaginal ejaculatory latency time; TEMPE, topical eutectic mixture for premature ejaculation.

## Behavioural therapy

The aim of behavioural therapy is the development of sexual skills over time, which allows the male to delay ejaculation, increase sexual confidence, and reduce performance anxiety or stress ^61^. There are two types of behavioural therapy: psychotherapy and physical techniques ^62^.

Psychotherapy uses counselling to identify and correct any interpersonal problems, which may have precipitated or be contributing to PE ^8, 62^. Physical therapies involve a variety of exercises to slowly increase the degree of genital stimulation over time, which allows the male to gain control over his ejaculation ^8, 62^. This includes ‘stop-start’, ‘squeeze’, ‘sensate focus’, and pelvic floor muscle rehabilitation ^8, 62^. The efficacy of these studies is difficult to assess, as their inclusion criteria do not always meet ISSM PE definitions; they include various types of treatments with either unreported or variable treatment duration ^8, 62^. In addition, the number of men receiving treatment is small; the method of IELT measurement and follow-up is limited. Despite these reservations, behavioural therapy appears to improve IELT when compared with placebo in two out of four studies in a recent systematic review ^62^. However, the picture is more mixed when behavioural therapy was compared with active drugs (that is, antidepressants); there was a trend towards improved IELT with active drugs or no significant differences ^8, 9, 62^. There is a need for more high-quality studies which are appropriately powered to demonstrate an effect using contemporary PE definitions. The combination of behavioural therapy with drug treatment (for example, DPX, chlorpromazine, paroxetine, and citalopram) has shown a significant but small improvement in outcome measures (that is, IELT) compared with the active drug alone in the three studies included in the systematic review ^62^. The outcome measures included IELT in two studies and a validated questionnaire in one study. The improvement in IELT was 0.5–1 minute. Cormio *et al*. published the results of their well-designed open-label prospective randomised study in 2015 ^63^. The inclusion criterion was based on the ISSM definition of PE. They compared outcomes of on-demand DPX with on-demand DPX plus behavioural therapy. Both groups had a significant improvement in IELT; however, the combination therapy resulted in a further 102- and 210-second increase at 12 and 24 weeks, respectively ^63^. In addition, 80% within the combination group no longer had PE at 24 weeks (versus zero in the DPX-alone group) based on the validated questionnaire PEDT.

## Topical eutectic mixture for premature ejaculation

Topical local anaesthetic agents are the oldest treatments for PE. However, they are often criticised for not being specifically optimised for PE, for being slow-acting, and for requiring the use of condoms to prevent partner transference and resultant partner hypoanaesthesia ^64, 65^. Topical eutectic mixture for PE (TEMPE, Plethora Solutions Ltd, Chalgrove, UK) was designed specifically for PE. It consists of 7.5 mg of lignocaine and 2.5 mg of prilocaine dissolved within a solution, which prevents penetration into the keratinised skin of the penile shaft ^66^. This results in a localised desensitising effect ^66^. The efficacy has been evaluated in two phase III placebo-controlled multicentre studies ^64, 66^. In total, 530 men with mainly LPE according to the ISSM definition were randomly assigned to TEMPE or placebo in 69 centres across Europe and North America ^64, 66^. A 6.3- and 4.6-fold increase in IELT was observed in both studies, respectively. There was also a significant improvement in the interprofessional education (IPE) domain scores in both studies ^64, 66^. The odds of achieving an IELT of over 1 minute and over 2 minutes were 9 times and 12.8 times greater with TEMPE compared with placebo, respectively ^66^. However, although after 3 months 90% of men receiving TEMPE achieved a mean IELT of over 1 minute, 54% in the placebo group achieved the same ^66^. This suggests a very strong placebo effect, which again calls into question how much we truly understand about this condition and controversially whether it exists as a true medical condition.

## Selective serotonin reuptake inhibitors

SSRIs inhibit axonal reuptake of serotonin from the synaptic cleft of central and peripheral serotonergic neurons by 5-HT transporters, resulting in enhanced 5-HT transmission and stimulation of post-synaptic membrane 5-HT _2c_ receptors ^19^. SSRIs may be used daily or on demand. A recent meta-analysis evaluated long-acting (paroxetine, citalopram, fluoxetine, and fluvoxamine) and short-acting SSRIs ^61^. Paroxetine was associated with the best efficacy, with an 11.6-fold increase in IELT in one study ^61^. The use of an on-demand regime has been associated with less ejaculatory delay ^67– 70^. Adverse effects are usually mild and include fatigue, yawning, nausea, and loose stools ^61^. ED and libido reduction have also been reported, though less frequently compared with men using the drugs for depression ^61, 71^. Men must be cautioned about SSRI withdrawal syndrome with abrupt drug cessation ^61^.

DPX is currently the only SSRI marketed specifically and licenced for the treatment of PE. It has the largest efficacy and safety database, having been studied in over 6,000 men in a variety of well-conducted, placebo-controlled trials ^64^. A recent meta-analysis of PE treatments revealed that DPX is associated with a 3- to 4.3-fold increase in IELT over baseline in three large placebo-controlled trials ^61, 64^. DPX has a good safety profile with side effects similar to those observed with other SSRIs ^61, 72^. In a large open-label observational study comparing DPX with alternative treatments for PE (including other SSRIs, condoms, and behavioural therapy), DPX had a higher incidence of adverse events (12% versus 8.95%) ^72^. The highest incidence of adverse events was observed in men over 65 using DPX and men 30–39 years old using other therapies ^72^.

Despite the high efficacy of SSRIs and associated low rate of adverse events, the proportions of men who stopped using DPX were 79.1% within 6 months and 90.1% at 2 years ^73^. The commonest reasons for discontinuation were the following:

- Disappointment that PE was incurable and tablets needed for every sexual encounter
- Side effects
- Perceived poor efficacy
- Search for alternative treatments
- Unknown ^73^

This suggests that more efficacious therapies are still required. Although similar complaints may be expressed by men with ED, ultimately the insertion of a penile prosthesis will allow a man to have sexual intercourse. Unfortunately, this is not the case with men with PE.

## Phosphodiesterase-5 inhibitors

PDE5is are the gold-standard first treatment for men with ED. However, their role in PE remains unclear. PDE5 is expressed in the prostate, vas deferens, and seminal vesicles and may have a physiological role in ejaculation ^74– 76^. The coexistence of ED and PE is common, occurring in up to 50% in some studies ^77– 80^. In addition, 33.3% of men may confuse the two conditions ^81^. This is not surprising. Men with ED require higher levels of arousal to attain an erection or may attempt to rush sexual intercourse prior to loss of their erection ^80^. Both of these situations will predispose them to a reduction in ejaculatory control and possible PE. Conversely, men with PE require a reduction in arousal to control ejaculation, which may worsen erectile function ^80^. Sexual dysfunction is also associated with performance anxiety, which may worsen erectile function ^13, 82^.

Most studies appear to suggest that the use of PDE5is may be useful in the treatment of PE ^83– 85^. However, they have been criticised for their poor methodology ^82^. Two systematic reviews have failed to support the use of PDE5is in the treatment of PE except where PE coexists with ED ^61, 82^.

## Management options: novel agents

### DA-8031

DA-8031 is a potent SSRI with high affinity and selectivity for the serotonin transporter ^86^. DA-8031 inhibits the expulsion phase of ejaculation by modulating the activity of the bulbospongiosus muscle in male rats ^86, 87^. In addition, it is associated with reduced serotonin transporter occupancy, confirmed on positron emission tomography of the rat brain ^87^. This was recently evaluated in the first human trial to assess safety and tolerability after a single dose in healthy men ^88^. Men were randomly assigned to receive either the active drug or placebo. The drug was well tolerated up to the 80 mg dose. The commonest adverse events were nausea and hypotension. The authors found that a dose of 120 mg was associated with prolongation of the QT interval on electrocardiogram ^88^. This is potentially a very exciting drug for the future, and further studies are awaited ( Table 5).

**Table 5.** Novel agents for the treatment of premature ejaculation.

Drug	Mechanism of action
DA8031	Selective serotonin reuptake inhibitor
Epelsiban	Oxytocin antagonist
IX-01	Oxytocin antagonist
Modafinil	Poorly understood: possible activation of central dopaminergic and serotonergic pathway Narcolepsy drug
Alpha-blockers	Inhibition at bladder neck Inhibition of seminal emission
Botulinum-A toxin	Unclear: may inhibit contraction of bulbospongiosus by blocking neural transmission

### Oxytocin antagonists

Oxytocin is a peptide hormone that is believed to shorten ejaculatory latency times through central and peripheral pathways in animal models ^8, 20, 89^. The ejaculatory delay observed with SSRIs may be mediated by alterations in the central release of oxytocin ^90, 91^. This has formed the basis of the development of oxytocin receptor antagonists. The first drug used in human clinical trials was epelsiban. This is a highly selective oxytocin receptor antagonist with rapid absorption and elimination ^20^. It had been shown to delay ejaculatory latency in animal models ^20^. In this multicentre study, men were randomly assigned to placebo or epelsiban (50 mg/150 mg) ^20^. Study inclusion was based on the ISSM definition of PE, and IELT was measured with the use of a stopwatch. There was no significant difference in the baseline IELT measurements between the groups. Following 8 weeks of treatment, there were no significant clinical or statistically significant differences in IELT between the groups. The authors speculated that efficacy may be linked to centrally acting oxytocin antagonist activity ^20, 92, 93^. There is some evidence from animal studies to support this hypothesis ^20, 92, 94^. This has led to the development of the most recent oxytocin antagonist, IX-01 ^89, 95^. This is a small molecule with good central penetration and rapid absorption with potent antagonism of the oxytocin receptor ^95^. A total of 88 men with LPE were randomly assigned to either placebo or active drug (400 mg/800 mg). Inclusion appeared to be based on ISSM criteria. Following 8 weeks of treatment, IX-01 resulted in a 3-fold increase in IELT (1.6 in the placebo group). This improvement was observed as early as 2 weeks after starting treatment. The drug was well tolerated with no serious or severe adverse events; 21% reported at least one adverse event (30% in the placebo group). The dropout rate was high: 21% with the active drug and 27% in the placebo arm. This is obviously encouraging news for the field, and we should expect larger trials involving a more diverse population.

### Modafinil

This is a drug used in the treatment of narcolepsy. Its mechanism of action is poorly understood but is thought to be via activation of central dopaminergic and serotonin pathways ^65, 96^. A sexual study in male rats has shown that the administration of D-modafinil resulted in a significant delay in ejaculation latency times ^97^. This effect may be mediated via an increase in serotonin release in the brain or spinal cord or an action on the dopamine system or both ^98– 103^.

Tuken *et al*. evaluated the use of modafinil 100 mg in 55 men with LPE ^104^. Although PEP domain outcomes were significantly improved, there was only a modest increase in IELT, from 25 to 50 seconds ^104^. A pre-clinical study is underway to investigate the short-acting modafinil d-isomer for the treatment of PE ^89^.

### α1-adrenoceptor antagonists

α1-adrenoceptor antagonists have a long history of use for the treatment of lower urinary tract symptoms (LUTS) with established efficacy. However, they are associated with documented side effects, which include ejaculatory dysfunction. They mediate ejaculatory dysfunction via two mechanisms:

1. Inhibition at the bladder neck, prostate, and urethra, resulting in retrograde ejaculation
2. Inhibition of seminal emission resulting in ejaculatory dysfunction ^89^

In a placebo-controlled study by Cavallini, alfuzosin and terazosin were associated with a 50% rate of ejaculatory delay ^105^. A similar study by Başar *et al*., using only terazosin, found that 67% of men reported ejaculatory delay ^106^. A major criticism of both of these studies was that neither used IELT to define efficacy. Efficacy was based on patient impression of change and sexual satisfaction ^89^. These two studies are relatively historic studies and their PE definitions are unlikely to be compatible with current accepted definitions.

Silodosin is a new, highly selective α1-adrenoceptor antagonist which has been associated with good efficacy for the treatment of LUTS but higher rates of ejaculatory dysfunction compared with other α1-adrenoceptor antagonists ^107– 110^. Sato *et al*. evaluated its use in the treatment of PE in a small prospective study of eight men ^111^. Inclusion criteria were based on the DSM-IV-TR definition. Efficacy outcomes were based on change in IELT and the PEP questionnaire domains. At the end of the 2-month treatment period, there was a significant improvement in IELT, from 3.4 to 10.1 minutes. In addition, all patients reported that their ejaculation problem was ‘much better’ or ‘slightly better’. Anejaculation (25%), reduced semen volume (37.5%), and pain during orgasm (87.5%) were common. This study offers an interesting and novel treatment for PE. However, there are several limitations. The most obvious limitation would be the use of the DSM-IV-TR definition, which allowed the inclusion of men who would not qualify as having PE according to the ISSM definition. This translated into a high baseline IELT at 3.4 minutes. In addition, 75% were on PDE5is, a known treatment modality for PE. In addition, there was a high rate of adverse events, which may correspond to a high dropout rate when translated into the typical young population with PE. Similar results were obtained in studies by Akin *et al*. ^112^ and Sato *et al*. ^111^, where silodosin was compared with other α1-adrenoceptor antagonists ^73^. Ideally, before it is possible to fully assess the efficacy of silodosin, there must be a placebo-controlled trial with a more diverse PE population using the ISSM criteria.

### Botulinum-A toxin

This neurotoxin is produced by the bacterium *Clostridium botulinum.* It selectively inhibits the release of acetylcholine from nerve endings, thereby blocking neural transmission when injected into muscle ^89^. It has revolutionised the management of detrusor instability. Serefoglu *et al*. have suggested that Botox may inhibit muscle contractions during the ejection phase of ejaculation when injected percutaneously into the bulbospongiosus muscles of male rats ^113^. This resulted in a significant increase in ejaculatory latency times. The effect peaked after 11 days and decreased sharply after 14 days. The difference in post-treatment ejaculatory latency between Botox and saline did not reach statistical significance.

There are currently patents for the use of Botox injected into the penis, frenulum, glans, and prepuce for the treatment of PE ^114^. This is both surprising and alarming in the absence of human trials and only minimal scientific evidence where Botox was injected into the bulbospongiosus muscles. However, a phase II clinical trial in humans is underway and we await its results ^89^.

## Conclusions

PE is a common problem with variability in definitions and efficacy measures of drugs used for this condition. The real importance for future drug development is the unification of definitions of PE and an agreed standard for what are acceptable measures and inclusion criteria in the trial setting. There remains uncertainty in assessing the true value of some of the drugs we already use in the management of this condition. There are different definitions for PE, different efficacy measures, and poor methodology in clinical trials. In addition, the high discontinuation rates suggest that our patients remain dissatisfied with the treatments we currently offer. Furthermore, one must be cautious to define a pathological condition often associated with a high placebo effect.

There are currently several novel agents which show promise for the future, but our optimism must be balanced against accepting suboptimal science and methodology in drug trials. PE continues to be the Cinderella of sexual medicine and requires funding and high-level research in both basic science and clinical trials. The oxytocin antagonists probably show the most future promise but data are still premature.
